# Surgical Reports

**Published:** 1877-11

**Authors:** Charles A. Wall


BUFFALO

### Medical and Surgical Journal.

VOL. XVII. NOVEMBER, 1877. No. 4.

### Original Communications.

———:o:———

ART. 1.—*Surgical Reports.* By CHARLES A. WALL, B. S.

The cases presented, are taken from the private practice of Prof. JULIUS F. MINER, and will be unaccompanied with remarks other than the views of the writer.

These are not clinical reports of cases brought before a class to illustrate any given disease, but those which occurred in the routine of professional duty; we cannot therefore give the Doctor's ideas upon the various operations, except perhaps in those exceptional cases where we have expressly sought his opinion in order to elaborate remarks upon a particular and interesting case. The reports will only contain those operations which we consider of interest to the profession at large, but no startling cures of extraordinary cases are to be expected; true reports of those actually seen can be relied upon as our aim, and the apology for writing the article.

Sequence as to time is not followed in the order of cases, since this would isolate those which naturally fall together.

SURGERY ANTISEPTIC.—We would first call attention to a case, which, if it had been treated after the manner of Lister, would have been thought proof absolute, of the benefit and surgical necessity of the use of the antiseptic dressing of wounds.

CASE I.—*Incised wound of knee and division of biceps.*—Wm. Baker, about twenty-four years of age, an employé of the Lake Shore R. R., was run over by a car about midnight of March 14th, 1877. He suffered injury of the knee, arm and foot. Dr. Miner found that a bolt or spike had penetrated the knee-joint gouging out a piece of bone from the internal condyle of the right femur and rupturing the ligaments of the joint to a slight extent. The finger could easily be passed beneath the patella. The piece of bone removed from the wound was an irregular cuboid of five-eighths of an inch. It was loose and was removed by the finger. The right arm had been run over, and the outer and posterior portion was badly lacerated, while the biceps had been severed subcutaneously at its middle. It presented two abnormal eminences, with an interval of three inches in which there was no muscular tissue between integument and humerus. Thinking these might be coagulated blood, the lower was cut down upon when healthy muscular tissue was exposed. No lesions of bone or arteries could be found in this region. The right foot had been jammed, the external metatarsal crushed in, or cuboid outwardly displaced, and when first seen greatly swollen. An effort was made to replace these bones but it was found impossible to entirely restore them. This, although seemingly the least important, was the most lasting of all his injuries.

As is the case after most accidents to railroad men, it was found that whisky had been freely administered by his attendants so that he was stupid, and did not seem to suffer to any considerable degree; but however, when upon a second visit some eighteen hours after injury he continued in a quiet, and apparently painless state, the query arose as to whether it was not the result of shock from which he was slow to rally. As time went on it was found that there was no change, and the conclusion was, that he had not been constitutionally affected to any considerable degree; indeed he only took a very few doses of anodyne, and those because he complained of pain in his left leg from one or two places, where the cuticle had been rubbed off.

The dressings were simple, consisting of compresses dipped in warm water and held to their places by a few turns of a bandage.

Whisky was ordered to be poured upon the cloths if the discharge became offensive. This is a favorite mode of dressing with the Doctor, who informs me that after numerous trials of the various methods he has found none affording better results. It is to be especially recommended in all cases where death of a part is to be feared. The warmth of the water acts as a stimulant to the circulation, often retaining life in members to the astonishment of the attending surgeon.

The arm and leg were each placed in a partially flexed position, a pillow being put under the knee. Synovia continued to flow from the wound at knee for a few days, when it healed and no further trouble was experienced. There was no suppuration whatever.

The injury to the anterior portion of the arm caused no trouble, while the lacerated wound on the posterior healed naturally and kindly. At no time was the pulse high or temperature perceptibly increased. I find the following in my note-book. March 15th. He lies quiet and does not complain; is not in pain; wounds look healthy; eats and sleeps as usual. 16th, 17th, and 18th. Remains in same condition as upon 15th; wounds are doing finely; no redness or other signs of inflammation about knee; muscular eminences upon arm not moveable as when first seen. 20th. Complains of cutaneous bruises on left leg, but has no pain in right arm or leg, except in foot upon pressure. 30th. Wound into knee is now completely healed; can flex leg to a great extent; arm nearly well; wishes to go out; moves from bed to lounge and back, with slight assistance. April 6th. Found the patient sitting in an easy chair with foot supported; goes around the house on crutches using two, the right arm doing equal service with left; says he could walk as well as ever if it was not for the trouble his foot gives him; has entire control over leg as to flexion and extension at knee. April 20th. When last seen he had walked over half a mile and had ridden three miles up to the office of Dr. Miner. He had not discarded his crutches since his foot troubled him when he bore his whole weight upon it. His arm was well, save the loss of complete extension, the biceps had shortened in uniting, so that extension was possible only to one hundred and sixty degrees, beyond this it could not be moved even by use of some force. He was dismissed cured, and soon after resumed work.

Such was the case, and such was the result. What was to be expected. Here was a wound penetrating the synovial sack at knee-joint, rupturing ligaments, and knocking a piece from the articular surface of the femur. The natural sequence is inflammation of the joint surface extending to adjacent structure, then anchylosis if not amputation or death. This is not placing too high an estimate upon the severity of this wound. Hamilton, Holmes and Bryant all agree in calling it one of those cases in which amputation is the only resort in military surgery, and authorized in many cases in private practice. Gross and Erichsen recommend immediately enlarging the wound, since it will be impossible to prevent extended suppuration. The prognosis is always exceedingly unfavorable. Anchylosis is to be hoped for as the best result. The German surgeons by Lister's method, report one-half of these cases cured, with moveable joints. Had this been a surgical wound made under the spray, and afterwards treated by the antiseptic method by a devotee to that idea, how it would have resounded through both old and new world. What more absolute and certain proof of its efficacy; what other case would be needed; is not this enough? Now we only see in it the power nature may in some instances exert in favor of poor humanity even outside of the hobby of some great thinker. To show to what an extent a man may be carried away by an idea of which he is possessed, we will quote Dr. Caldwell, in a communication to the *Chicago Medical Journal and Examiner* upon Mr. Lister and his antiseptic method. After speaking of his removal to King's College, London, he says: "As the school to which he goes is much smaller than the one here, and the number of beds of which he will have charge fewer than those he here controls, some surprise has been expressed by his friends at his accepting his new position. His reply is that he hopes in London, to be able to diffuse more widely his views on the germ theory of the contamination of open wounds after surgical operations."

Lister claims that the reason so many fail in their results, is owing to lack of knowledge or care in attempting to practice antiseptic surgery, and Dr. C. by an illustration, which the *Louisville Medical News* ironically quotes under the head, "*A Brutal Murder*," shows Lister's views. "To illustrate the extreme care that is necessary in the practice of antiseptic surgery, he related to me the following case. He was opening under the spray a psoas abscess, the result of disease of the spine. His assistant who was working the spray apparatus, becoming annoyed at a student behind him, and kicking at him, allowed the jet of spray for just an instant to be so directed as not to cover the opening he had just made. In great anger the Doctor told his assistant that if that patient died he might consider himself the cause of her death. She did die, and Mr. Lister believes that the misdirection of that jet of spray for the small fraction of a minute was the cause of her death." Can Mr. Lister blame New York surgeons, even if they do as he says, cry 'humbug,' and question if his results are due to his methods. For ourselves it seems nonsense to claim any power for an agent, which destroying septic poison isolated from tissue is unable to affect it when it is even for an instant past in contact with living organization. If it has power, and we do not wish to be understood as denying it, will it not be able to affect as much at one time as another, or does it fail to act if brought within the modifying power of the exposed wound? These are Mr. Lister's views; septic poison is a bird of prey to which carbolic acid is a deadly bolt if shot upon the wing, but woe be unto the patient if it is allowed to settle, for suppuration and death follow in its track. We know that Mr. Lister accounts for this upon the idea that the contaminating germ has penetrated the wound beyond the reach of the spray. We ask, why does not the latter follow and destroy the former? By the method employed the spray is reduced to the magnitude of the germ. Will it not penetrate as far? Mr. Lister answers no, and employs the tissue destroying agent chloride of zinc and annihilates the germ at the expense of living cells.

If Mr. Lister has results more gratifying than other operators they are to be accounted for without giving his disinfectant all the credit. By his manner of procedure, three important objects are attained. First, since every ligature, sponge, knife or forceps is washed in carbolized water immediately before use, they are thus in as clean and uncontaminated a state as possible. No one can afford to secure new each time, and therefore the next best thing is to thoroughly cleanse the old. Second, his way of operating is careful and scientific. He has caught the right idea, when he says that except in rare cases, all surgical operations should be performed in the most slow and deliberate manner. And lastly, the utmost cleanliness is observed, when the dressings are changed, the wound being washed with carbolized water under carbolic spray. While the carbolic acid may or may not be of use as a destroyer of germs the washing are undoubtedly of great value, simply in freeing the wound of dead or foreign matter. Carbolic acid has its uses since as a disinfectant it is unequalled, but the odor is so unpleasant to some that it cannot be tolerated. Another disinfectant must then be substituted. In cases of unpleasant and disagreeable discharge, nothing is of more value; it must ever retain a high place in the opinion of the profession from this quality; and thus is it of importance no matter what its effects may be upon germ life. Mr. Lister may live to see the day, when the universal verdict will have been pronounced, that carbolic acid, no matter how assiduously used, will not in every case, even under the most favorable circumstances be a guarantee of perfect cure, or assure freedom from suppuration. What mode of dressing wounds can yield better results than those of Alanson, of Liverpool, who in 1779, amputated the thigh 33 times without a single death?

Case II.—*Compound Fracture within two inches of ankle joint.* April 26th, 1877. Dr. Miner was called to see Mr. S., aged 36, who in jumping from a wagon had fractured both tibia and fibula within two inches of the ankle. Falling as he did upon the inner side of the foot the force had been sufficient to project the tibia through the integument and the side of his shoe. The external maleolus had been fractured and the bone driven down on the side of the tarsus. When seen, the foot lay at right angles to the tibia, which extended through the skin an inch and a half, although the shoe had been previously removed, and an endeavor made to bring the foot into place. There had been considerable ligamentous and muscular disturbance, but no arterial lesions were found; little hemorrhage occurred. Three quarters of an inch of the projecting bone having been cut off the foot was brought into line with the leg, and side splints applied, the wound being dressed with cloths wrung out of hot water. The outer splint was heavily padded upon the lower portion in order to invert the foot to the greatest possible extent. The difficulty in fractures of the bones of the leg at the lower end, is the tendency to turn out. The peroneal muscles evert the foot, and require considerable lateral force to overcome them. Extension cannot be used from the impossibility experienced in attaching and retaining adhesive strips; they become loose after a few hours. Plaster of Paris bandages are often of great value. The testimony of most surgeons is that with the best care more or less eversion will result.

The foot was dressed without the aid of an anæsthetic. Opium was ordered if in pain. April 27th. The splints had not been able to keep the foot in position; it was badly everted. They were removed, the outer more heavily padded and again applied. It seemed impossible now for the foot to move, but when next visited it was as before, the tendency to turn out had overcome the resistance, and deformity was too apparent to remain unnoticed even by his attendants, who sent in haste for the Doctor to come and reset the fracture. The inner splint was removed and a compress having been laid upon the tibia the leg was again dressed. This became so painful before night that he was obliged to send for Dr. Miner in order to have it changed. The compress was removed, but inserted upon the next visit, and retained in place without causing pain until the 30th, when it was of so much trouble to him that it was removed. A base splint together with the outer side one was put on, and remained with few exceptions, until the first of June, one or the other being removed for a day or two at various times, as it became unbearable. For the first three weeks every visit found it more or less everted, so that a day did not pass without the necessity for dressing it. The fracture was so low down, and the inflammation so great that extension was out of the question, it could not be applied; it was not until the middle of May, when the fibula united that any success was experienced in keeping the foot from day to day in its proper position. May 2d. An abscess on the anterior of ankle was opened and considerable sphacelated muscular tissue removed, this soon healed, but another formed on the outer side of leg, and freely discharged for a week. May 30th. The wound on the inner side did not heal over and the tibia was rough and denuded of periosteum for an inch and a half, so the patient, having been brought under the influence of an anæsthetic, Dr. Miner operated upon the leg, removing all the dead and denuded bone. This was easily accomplished, since the line of junction between the live and dead bone was plainly marked. In a few days the wound was entirely healed. About the end of the seventh week after the injury, suppuration ceased and he began to go about upon crutches. The result is as good as could be expected after so serious an injury. At no time was he constitutionally affected to any great extent. The leg is now, Sept. 24th, one and one half inches short; there is motion in the ankle, which is increased in size. The external maleolus had been forced downward and outward. He has a shoe made to accommodate this enlargement, the sole also being thicker than that on the right shoe. He walks without the assistance of crutches or cane. It is not sensitive when used; the direction is perfect, the foot neither turning inward or outward. This was not dressed antiseptically; an oil meal poultice was substituted for the warm water dressings when the discharge was profuse; it served as a receptacle for the discharged pus, and did not require changing as often as the latter.

CASE III.—*Compound Fracture—Amputation.*—April 25th. Dr. Miner was called in haste to attend Wm. B., aged 27, who in falling from a wagon had caught his right foot between the spokes of a wheel, and fractured the tibia and fibula within an inch or two of the ankle joint. The lower end of tibia had been badly comminuted, and forced through the skin. After the patient had been brought under the influence of ether, Dr. Miner enlarged the wound and removed eight or ten pieces of bone, but when it was found that the anterior and posterior tibial and peroneal arteries had all been severed, amputation was determined to be the only resort. The leg was accordingly removed at the junction of the middle and lower third by anterior and posterior flap. The patient, after having the leg dressed with warm water was then placed upon his bed and allowed to come out from under the anæsthetic.

Anodyne was given him in small quantities to quiet restlessness. On the fifth day after operation, the patient was handed over to the family physician. At no time did he suffer constitutionally; the stump healed kindly and rapidly although not by first intention. About the middle of May, the patient's brother came into the office and reported him well. The foot was dissected after removal, when it was found that not only were the lower ends of the tibia and fibula much shattered, but the os cuboid and astrangulus were broken into numerous pieces, and the ligaments of the joint badly ruptured. So great had been the disturbance that if the arterial supply had remained intact, no hope could have been entertained of saving the foot. The ankle joint was entirely destroyed, the surfaces of bone in aposition being ground into powder.

Case IV.—*Psoas Abscess opened.—Recovery.* April 8th, 1877, Dr. Miner was called to see Dan M., aet. 11, who had been suffering for two or three months with pain in his back and side. Latterly a bunch had appeared in the left inguinal region. For a week past the patient had been confined to his bed. His left leg was drawn up over his abdomen, and he was obliged to lie upon his right side. There was considerable tenderness to pressure over the entire left side of abdomen without any distinct pointing of the abscess. Warm water dressing were ordered. Dr. Miner determined to open it at his next visit, but could not persuade the boy to allow it until the 16th, when an anæsthetic was administered notwithstanding his struggles. The abscess was then opened and pus to the amount of about four ounces discharged. The abscess was deeply situated, although considerable time had been allowed that it might point and be the more readily opened. The discharge of pus continued for about two weeks and then ceased, and the puncture healed; no ill effects have since manifested themselves. The result of opening the abscess was magical. While before this the patient conld only obtain rest from the influence of an anodyne, afterwards within two days he slept for six hours without its use. His leg straightened and in four days he was around the house making use of a cane to support himself. He immediately began to gain in flesh, and when last seen, two months after operation, was as large and healthy as any boy of his age.

The dressing applied was warm water to which a few drops of whisky had been added to cover the offensive odor. Lister claims that the grandest results of his method are witnessed in surgical wounds, and we have already referred to an illustrative case in which the spray, for only a few seconds, had been misdirected, causing, as he says, death. How many opportunities were open for septic poison to enter the surgical wound on Dan M., and still he lives. We must conclude then that asepsis by the use of carbolic or salicylic acid is not of paramount and critical importance since recovery often follows without its exhibition. Good or bad results will follow from any mode of dressing; none will assure a given result, and Lister must grant his theory still unproven.

We quote from the eminent surgeon T. Spencer Wells, in his address before the British Medical Association. "For my own part, I would rather operate in a clean, quiet, well warmed, and well ventilated building, be it large or small, without any antiseptic precautions, than run the risk of trusting the neutralizing or destructive power of chlorine or iodine, sulphur or tar, borax or the permanganates, salicylic or any other acid, in a place tainted by the presence of sewer gas or the seeds of some infectious or contagious disease." We present the above to show the value of cleanliness, and the care needed to surround the patient with every favoring circumstance. We will close this article with another quotation taken from the *Medical and Surgical Reporter*. "Many surgeons maintain that the success of Lister's antiseptic treatment is more owing to the care and cleanliness he inculcates than to anything else. The following extract from a Paris letter, shows how much these are wanting in the hospitals of that city, 'at one of the best known hospitals in Paris, I witnessed the excision of a fatty tumor, the operation being as simple a one as possible. The man was put to bed, and next day a case of facial erysipelas was taken into hospital and deliberately placed in the next bed, needless to say, the patient who had been operated upon took the disease and died of it. In another hospital a case of erysipelas was introduced from outside and placed between two patients who were convalescent from acute diseases, they both took the disease and died, and in addition several other patients in the same ward took the disease. As no disinfection or isolation was practiced, it was long before the ward was free from the infection; these cases are only examples of what is taking place almost every day in Paris. It is really incredible that such a state of things should exist in a great town like Paris, which prides itself upon being the home of surgery, and boast that it has some of the finest surgeons of the world.'"

